# Medicaid Expansion and State-Level Differences in Longevity-Based Aging Thresholds

**DOI:** 10.1093/geroni/igaf122.4388

**Published:** 2025-12-31

**Authors:** Arun Balachandran, Danny Alex, Daniel Belsky

**Affiliations:** Columbia University, New York City, New York, United States; Florida State University, New York, New York, United States; Columbia University, New York, New York, United States

## Abstract

The Affordable Care Act (ACA), implemented in 2014, allowed U.S. states to expand Medicaid eligibility to previously uninsured populations. The timing and extent of expansion varied across states. While prior research has examined ACA-related mortality outcomes among younger adults, its long-term effects on older populations remain underexplored. This study examines how Medicaid expansion influenced longevity and wellbeing among older adults using two novel demographic indicators: the Prospective Old Age Threshold (POAT) and the Comparative Prospective Old Age Threshold (CPOAT). POAT represents the age at which remaining life expectancy equals 15 years, while CPOAT adjusts this measure to reflect state-level variation in the rarity of reaching POAT. Both measures capture shifts in the “old-age threshold,” with higher thresholds indicating greater gains in life expectancy. We analyzed state-level demographic and mortality data from 2010–2022 linking national datasets, including CDC WONDER, the Behavioral Risk Factor Surveillance System, the Health Resources and Services Administration, the U.S. Census Bureau, and the Bureau of Labor Statistics. States were classified as Medicaid expansion or non-expansion based on Kaiser Family Foundation records. Descriptive analyses and multivariate difference-in-differences models were used to assess annual state-level changes in aging thresholds. Preliminary findings indicate that Medicaid expansion was associated with larger increases in POAT and CPOAT (∼1.8 to 2.4 years), with stronger effects in states that expanded earlier. Although the magnitude varied across measures and covariates, the overall trend suggests Medicaid expansion improved longevity among older adults. These findings underscore Medicaid expansion’s role in promoting equitable health gains across aging populations.

